# A brief dementia screener suitable for use by non-specialists in resource poor settings—the cross-cultural derivation and validation of the brief Community Screening Instrument for Dementia

**DOI:** 10.1002/gps.2622

**Published:** 2010-12-28

**Authors:** M Prince, D Acosta, C P Ferri, M Guerra, Y Huang, K S Jacob, J J Llibre Rodriguez, A Salas, A L Sosa, J D Williams, K S Hall, the 10/66 Dementia Group

**Affiliations:** 1 Centre for Global Mental Health, Health Service and Population Research Department, King's College London, Institute of PsychiatryUK; 2 Internal Medicine Department, Geriatric Section, Universidad Nacional Pedro Henriquez Ureña (UNPHU)Santo Domingo, Dominican Republic; 3 Universidad Peruana Cayetano Heredia and Instituto de la Memoria y Desordenes RelacionadosLima, Perú, UK; 4 Key Laboratory of Mental Health, Ministry of Health (Peking University), Peking University Institute of Mental HealthBeijing, China; 5 Christian Medical CollegeVellore, India; 6 Facultad de Medicina Finley-Albarran, Medical University of HavanaCuba; 7 Medicine Department, Caracas University Hospital, Faculty of Medicine, Universidad Central de VenezuelaCaracas; 8 The Cognition and Behavior Unit, National Institute of Neurology and Neurosurgery of Mexico, Autonomous National University of MexicoDelegacion Tlalpan, Mexico City, UK; 9 Voluntary Health ServicesChennai, India; 10 Department of Psychiatry, Indiana University Medical SchoolIndianapolis, Indiana, USA

**Keywords:** dementia, diagnosis, screening, cognitive test

## Abstract

**Objective:**

Brief screening tools for dementia for use by non-specialists in primary care have yet to be validated in non-western settings where cultural factors and limited education may complicate the task. We aimed to derive a brief version of cognitive and informant scales from the Community Screening Instrument for Dementia (CSI-D) and to carry out initial assessments of their likely validity.

**Methods:**

We applied Mokken analysis to CSI-D cognitive and informant scale data from 15 022 participants in representative population-based surveys in Latin America, India and China, to identify a subset of items from each that conformed optimally to item response theory scaling principles. The validity coefficients of the resulting brief scales (area under ROC curve, optimal cutpoint, sensitivity, specificity and Youden's index) were estimated from data collected in a previous cross-cultural validation of the full CSI-D.

**Results:**

Seven cognitive items (Loevinger H coefficient 0.64) and six informant items (Loevinger H coefficient 0.69) were selected with excellent hierarchical scaling properties. For the brief cognitive scale, AUROC varied between 0.88 and 0.97, for the brief informant scale between 0.92 and 1.00, and for the combined algorithm between 0.94 and 1.00. Optimal cutpoints did not vary between regions. Youden's index for the combined algorithm varied between 0.78 and 1.00 by region.

**Conclusion:**

A brief version of the full CSI-D appears to share the favourable culture- and education-fair screening properties of the full assessment, despite considerable abbreviation. The feasibility and validity of the brief version still needs to be established in routine primary care. Copyright © 2010 John Wiley & Sons, Ltd.

## Introduction

Early dementia diagnosis is currently actively promoted in developed countries, with a view to preparing those affected, and their carers, and to ensure timely access to support and care when it is needed (Department of Health, 2009). In low and middle income countries (LMIC), dementia remains to a large extent a hidden problem. Although the symptoms and syndrome are widely recognized and named, it is considered to be a normal part of ageing, not a medical condition (Cohen, 1995;Patel and Prince, 2001; Shaji *et al.*, 2002a, 2002b). Family members rarely seek help, and primary care doctors rarely come across cases (Patel and Prince, 2001; Prince *et al.*, 2007a, 2007b). The treatment gap in south India was recently estimated to be as high as 90% (Dias and Patel, 2009). Nevertheless, dementia is an important source of carer strain (Prince *et al.*, 2007a, 2007b). In LMIC, community-based career education and training interventions have recently been shown to be particularly effective in reducing career strain and depression (Dias *et al.*, 2008; Gavrilova *et al.*, 2008). The World Health Organization is preparing evidence based guidelines for management of dementia by non-specialists in LMIC with a view to scaling up treatment and reducing the treatment gap (World Health Organization, 2008), and evidence-based packages of care have recently been proposed (Prince *et al.*, 2009). Effective case identification by non-specialists is an essential element (Prince *et al.*, 2009).

Studies in high income countries (HIC) show that only a fifth to a half of cases of dementia are routinely recognised and documented in primary care case note records; with a median proportion from six studies of 39% (Olafsdottir *et al.*, 2000; Valcour *et al.*, 2000; Lopponen *et al.*, 2003; Boustani *et al.*, 2005; Wilkins *et al.*, 2007). However, evidence suggests that primary care physicians and nurses can, if specifically prompted to do so, make a dementia diagnosis with reasonable accuracy, using their knowledge of the patient, available case note information and their own routine assessments in the limited time available during a typical consultation (O'Connor *et al.*, 1988; Cooper *et al.*, 1992). Similarly, in LMIC community healthcare workers could, with a few hours training, identify dementia in the community with a positive predictive value of 66%, based solely upon their prior knowledge of older people from their routine outreach work (Shaji *et al.*, 2002a, 2002b; Ramos-Cerqueira *et al.*, 2005). The discrepancy between what non-specialists might, and do in practice achieve is explained partly by limited help seeking. It may also be that non-specialists either do not attend to dementia, or are not motivated to confirm and record the diagnosis when the possibility occurs to them.

Population screening for dementia is not considered cost-effective even in HIC (National Collaborating Centre for Mental Health, 2007). However, indicated screening of primary care attendees, based upon prior suspicion of possible dementia, can promote case detection. Research in developed countries has highlighted the short period of time available for each consultation in primary care, and the need accordingly for very brief assessments, ideally taking 5 min or less to complete (Brodaty *et al.*, 2006). Screening involves cognitive testing of the older person or informant interview for a history of cognitive and functional decline. Sometimes both approaches are combined in a single test. The Mini-Mental State Examination (Folstein *et al.*, 1975) is widely used in HIC, and adapted versions have been developed for use in many LMIC (Ganguli *et al.*, 1995; Xu *et al.*, 2003; Castro-Costa *et al.*, 2008). However, it takes 10 min to administer and is prone to educational and cultural bias (Black *et al.*, 1999; Ng *et al.*, 2007). A brief version of the MMSE, the ‘six item screener’ performed as well as the full MMSE in clinical and population samples in the USA (Callahan *et al.*, 2002). The three tools that are brief enough, and at least as valid as the longer MMSE (General Practitioner Assessment of Cognition (GPCOG (Brodaty *et al.*, 2002)), the Memory Impairment Screen (MIS (Buschke *et al.*, 1999)) and Mini Cog (Borson *et al.*, 2000)) have only been validated in HIC (Brodaty *et al.*, 2006). Reviewing their content, none is suitable for use in low education LMIC settings. MIS requires reading ability, and GPCOG and Mini Cog include clock drawing tasks that are not generally feasible for those with less than 5 years education (Lessig *et al.*, 2008). The adaptation required to make them suitable would, in effect, be equivalent to the development of a new assessment. The recently developed Vellore Screening Instrument for Dementia seems promising, but with 10 cognitive and 10 informant items it may be too long for routine use. Furthermore, its only community validation to date was on a sample of only 101 participants, three of whom were diagnosed with dementia (Stanley *et al.*, 2009).

The Community Screening Instrument for Dementia (CSI ‘D’) (Hall *et al.*, 1993) is by far the most extensively validated dementia screening assessment, across a variety of LMIC. It combines culture and education-fair cognitive testing of the participant (32 items) and an informant interview enquiring after the participant's daily functioning and general health (26 items) into a single predictive algorithm. It was developed and first validated among Cree American Indians (Hall *et al.*, 1993; Hendrie *et al.*, 1993), and further validated and used in population-based research among Nigerians in Ibadan and African-Americans in Indianapolis (Hendrie *et al.*, 1995). It has also been validated in Jamaica and among white Canadians in Winnipeg, (Hall *et al.*, 2000). The CSI-D test score distributions among those with dementia and controls, and the degree of discrimination provided were remarkably consistent across these five very different cultural settings (Hall *et al.*, 2000). CSI ‘D’ was further validated in the community among 2885 persons aged 60 and over recruited in 25 centres in India, China and South East Asia, Latin America and the Caribbean and Africa, as part of the 10/66 Dementia Diagnosis Protocol (Prince *et al.*, 2003). The inclusion of the informant interview significantly improved upon the predictive power of the CSI ‘D’ cognitive test component (Hall *et al.*, 2000; Prince *et al.*, 2003). However, requiring around 30 min to administer, it is too long for routine use in primary care. It could, in principle, be shortened. Adoption of a simple scoring method will also add to its value as a primary care screening assessment. The purpose of the secondary analyses presented in this paper, using existing data from the 10/66 Dementia Research Group pilot studies and population-based studies, is to explore the potential for deriving much briefer cognitive and informant scales from the full CSI-D and to carry out initial assessments of their likely validity.

## Methods

Three 10/66 Dementia Research Group data sets were used in this analysis, one to develop brief versions of the CSI-D cognitive and informant interviews with favourable scaling properties, and two to test their likely validity against an independent gold standard dementia diagnosis.

### Development data set, and item reduction procedures

Data from 15 022 participants in the 10/66 Dementia Research Group population-based studies in 11 sites in Latin America (Cuba, Dominican Republic, Mexico, Peru, Venezuela), India and China were used to carry out an item reduction of the CSI-D cognitive and informant interviews, based on conformity with item response theory (IRT) principles, the aim being to identify a small number of items with strong hierarchical scaling properties. The survey protocol is described in detail elsewhere (Prince *et al.*, 2007a, 2007b). In the course of the assessment comprising clinical interview, cognitive assessment, physical examination and informant interview, the full CSI-D cognitive test and informant interview were administered to the participant and an informant who knew them well (usually a co-resident family member). Participation was by informed signed consent, or signed witnessed verbal consent in the event of illiteracy, or signed assent from a relative in the event of lack of capacity; ethical approval was obtained from the King's College London Research Ethics Committee, and from the locally responsible institutional review board in each site. Mokken analysis was used to select a brief subset of CSI-D items representing an adequate hierarchical scale, separately for cognitive and informant items, using the Stata program LoevH. Mokken scaling involves the application of a non-parametric item response model (Mokken, 1971) to measure the hierarchical properties of items in a scale, assessing if the items can be ordered by degree of difficulty, so that any individual who endorses a particular item will also endorse all the items ranked lower in difficulty. Three basic assumptions are required for a monotone homogeneity model (MHM): 1) unidimensionality (one latent variable summarises the variation in the item scores in the questionnaire), 2) local independence (after conditioning on the position on the latent trait, the item scores are statistically independent) and 3) monotonicity (for all items the probability of a positive response increases monotonically with increasing values of the latent trait). These assumptions being met, an individual's position on the latent trait can conveniently be estimated as the rank of the highest item in the hierarchy that they endorse, or their total number of positive responses (Dijkstra *et al.*, 1999). Double monotonicity models (DMM) require in addition that for any value of the latent trait, the probability of a positive response decreases with the difficulty of the item. This means that the order of item difficulties remains invariant over all values of the latent trait and thus, that the item response function curves do not intersect (Van der Ark *et al.*, 2007; Sijtsma *et al.*, 2008). To assess single monotonicity, we estimated Loevinger coefficients for each item (H*i*) and for the whole scale (H), where values between 0.3 and 0.4 suggest weak scalability, values between 0.4 and 0.5 moderate scalability, and values above 0.5 strong scalability. We also tested formally for violations of monotonicity (using the Stata loevH monotonicity command) and non-intersection (using the Stata loevH nipmatrix command) between pairs of items (minimum violation 0.03, α = 0.05), using overall criteria values as an indication of the likelihood of assumption violation; ≤40 ‘satisfactory’, 40 to 79 ‘questionable violation’, 80 and over ‘strongly suggesting an assumption violation’ (Molenaar and Sijtsma, 2000). The subset of six items with the highest H*i* was selected for inclusion in the scale. Where more than six items offered strong scalability, priority was also given to selecting those with differing item difficulties, and representing different domains of cognitive function or disability. We also used IRT principles to simplify the scoring and scaling of the informant CSI-D by converting polytomous item responses (whether problems were observed to occur never, sometimes or often) into optimal dichotomies.

### Independent validation of the brief versions of the CSI-D

The performance of the draft brief versions of the cognitive and informant scales was then tested in two data sets in which dementia diagnosis had been made independent of the CSI-D assessment. In the 10/66 DRG pilot studies (Prince *et al.*, 2003) in 26 LMIC sites in Latin America, China and SE Asia, and Africa (see appendix for details), an independent clinician used their routine clinical assessments appropriate to the setting and culture, anchored around a clinical checklist proforma, and completion of the Clinical Dementia Rating (CDR) (Morris, 1993) to identify a case group with DSM-IV dementia of mild (CDR = 1) to moderate (CDR = 2) severity. Participants were also recruited into one of three control groups one with high education and no dementia, one with low education and no dementia and one with depression (not included in this analysis). The CSI-D was subsequently administered to all participants by a research worker, masked to knowledge of group status. Consent/assent arrangements were the same as those used for the population based studies (see above); ethical approval was obtained from the King's College London Research Ethics Committee, and from the locally responsible institutional review board in each site. In the 10/66 Dementia Research Group population-based study in Cuba, local clinicians (psychiatrists, geriatricians or physicians) administered the 10/66 survey interview, including the full cognitive and informant CSI-D, at the end of which they made their own diagnosis of clinically relevant dementia, guided by DSM-IV criteria (Prince *et al.*, 2008). They were at that time masked to the survey 10/66 Dementia diagnosis, which was generated later by applying a computerised algorithm.

For both data sets, brief cognitive and informant scores were calculated by summing the scores for the reduced set of items. An a priori decision was taken to generate a combined overall score (possible range −6 to 9) by subtracting the informant score (possible range 0 to 6) from the cognitive score (possible score 0 to 9). Subtraction, rather than addition was appropriate since lower scores on the cognitive scale but higher scores on the informant scale indicated impairment.

Validity was tested separately for centres from the four pilot study regions (Latin America, India, China and Nigeria), and for the Cuban population-based study sample. In a receiver operating curve (ROC) analysis sensitivity was plotted against 1-specificity and the area under the curve calculated, with 95% confidence intervals. The ROC analysis was used to identify the optimum cutpoint for each of the cognitive test, informant and combined brief scale scores, and sensitivity, specificity and Youden's index (Youden, 1950) (1-[sensitivity + specificity]) was calculated at this cutpoint.

## Results

The seven cognitive test items selected for inclusion in the brief scale were, in order of item difficulty; correctly describing the use of a hammer, naming elbow, pointing to the window and then to the door, locating the nearest store, orientation to season, orientation to day of the week, and delayed recall of three words (see Appendix for details of these items). The six informant items selected were, in order of item difficulty; often forgetting where she/he had put things, general decline in mental functioning, change in ability to think and reason, sometimes forgetting what happened the day before, sometimes forgetting where she/he is, and any difficulty dressing. For the brief cognitive scale the item level Loevinger coefficient (H*i*) varied between 0.59 and 0.79, while that for the scale as a whole (H) was 0.64. There were no violations of monotonicity or non-intersection assumptions. For the brief informant scale the item level Loevinger coefficient (H*i*) varied between 0.66 and 0.72, while that for the scale as a whole (H) was 0.69 (Table 1). Again, there were no violations of monotonicity or non-intersection assumptions.

**Table 1:** Mokken scale (item response theory) analyses for the development of the Community Screening Instrument for Dementia (CSI-D) brief informant and cognitive scales

	*n*	Item difficulty	Observed Guttman errors	Expected Guttman errors	Loevinger H coefficient	z-statistic	*p*-value
Cognitive scale							
Describing the use of a hammer	15 022	0.024	486	2289.2	0.79	73.2	<0.00001
Naming elbow	15 022	0.032	1105	2933.0	0.62	64.7	<0.00001
Pointing to window and then to door	15 022	0.040	1264	3457.8	0.63	69.9	<0.00001
Locating nearest store	15 022	0.059	1679	4423.4	0.62	73.1	<0.00001
Orientation to season	15 022	0.062	1793	4523.7	0.60	71.3	<0.00001
Orientation to day of the week	15 022	0.100	2210	5328.3	0.59	66.2	<0.00001
Delayed recall of three words	15 022	0.283	2597	7570.0	0.66	62.1	<0.00001
Whole Scale	15 022	-	5567	15262.7	0.64	116.3	<0.00001
Informant scale							
Often forgets where she/ he had put things	14 922	0.523	2334	7039.5	0.67	90.4	<0.00001
General decline in mental functioning	14 922	0.868	1833	5459.0	0.66	101.4	<0.00001
Change in ability to think and reason	14 922	0.908	1638	5297.7	0.69	117.2	<0.00001
Sometimes forgets what happened the day before	14 922	0.913	2143	7117.2	0.70	124.7	<0.00001
Sometimes forgets where she/ he is	14 922	0.968	1201	4291.7	0.72	108.3	<0.00001
Difficulty dressing	14 922	0.982	1105	3605.7	0.69	81.2	<0.00001
Whole scale	14 922	-	5127	16405.4	0.69	174.9	<0.00001

For the brief cognitive scale, areas under the ROC curve (AUROC) varied between 0.88 and 0.92, other than in the small Nigerian pilot study validation sample, where AUROC was 0.97. Optimal cutpoints were the same for all regions; five or less (favouring specificity over sensitivity) or six or less (favouring sensitivity over specificity). For the lower cutpoint Youden's index varied between 0.63 and 0.75 (0.92 in Nigeria). For the brief informant scale AUROC varied between 0.92 and 0.97, with a perfect AUROC of 1.00 in Nigeria. The optimal cutpoint was a score of two or more for all regions other than Nigeria (three or more). Youden's index varied between 0.70 and 0.88, but was 1.00 for Nigeria. Combining the cognitive and informant scores, by subtracting the informant score from the cognitive score yielded improved AUROC, ranging from 0.94 to 0.99, and 1.00 for Nigeria. Optimal cutpoints were again similar for all regions; four or less for all of the 10/66 pilot study regions, and the same for the Cuban population-based study sample if specificity was prioritised over sensitivity. Youden's index varied between 0.78 and 0.88, but was 1.00 for Nigeria (Table 2).

**Table 2:** The validity of the brief Community Screening Instrument for Dementia (CSI-D) informant scale, cognitive scale and combined algorithm, by region

Region	AUROC	Optimal cutpoint	Sensitivity (%)	Specificity (%)	Youden's index
	Brief CSI-D informant scale				
India	0.97 (0.94–0.99)	>1	87.9	99.7	0.88
China	0.96 (0.93–0.98)	>1	82.3	98.3	0.81
Latin America	0.96 (0.95–0.97)	>1	94.7	92.6	0.87
Nigeria	1.00 (1.00–1.00)	>2	100	100	1.00
Cuba (population)	0.92 (0.90–0.94)	>1	76.4	93.1	0.70
	Brief CSI-D cognitive scale				
India	0.91 (0.88–0.93)	<6/<7	72.4/86.5	93.5/81.8	0.66/0.68
China	0.92 (0.88–0.95)	<6/<7	81.3/94.5	95.1/67.4	0.75/0.62
Latin America	0.90 (0.88–0.92)	<6/<7	76.8/89.1	92.0/75.2	0.69/0.64
Nigeria	0.97 (0.94–1.00)	<6/<7	95.0/100.0	97.4/97.2	0.92/0.97
Cuba (population)	0.88 (0.85–0.90)	<6/<7	70.3/85.8	92.6/69.2	0.63/0.55
	Brief CSI-D combined scale				
India	0.98 (0.90–0.99)	<5	97.3	90.5	0.88
China	0.99 (0.98–1.00)	<5	93.5	94.4	0.88
Latin America	0.97 (0.97–0.98)	<5	89.0	93.4	0.82
Nigeria	1.00 (1.00–1.00)	<5	100	100	1.00
Cuba (population)	0.94 (0.93–0.96)	<5/ <6	85.1/92.1	92.4/85.2	0.78/0.77

Combining pilot study data across regions, the sensitivity of brief CSI-D was slightly lower for the detection of mild dementia than for moderate dementia – 69.2% compared with 82.8% for the cognitive score <6 cutpoint, and 90.3% versus 95.0% for the combined score <5 cutpoint. Likewise, specificity was slightly lower among low education than high education controls – 88.9% compared with 97.4% for the cognitive score <6 cutpoint, and 89.9% compared with 95.0% for the combined score <5 cutpoint.

## Discussion

This study has several strengths. To avoid over-estimation of validity coefficients from data-driven test development, the brief CSI-D was developed using one set of data, from large population-based surveys in Latin America, India and China, and tested on two others. The selection of the items for the abbreviated cognitive and informant scales was based solely upon optimisation of the efficient scaling of the underlying traits, through the application of IRT principles, without regard to their ability to discriminate between those with and without dementia. The seven items constituting the brief cognitive scale and the six brief informant scale items had very strong hierarchical properties. The brief CSI-D cognitive items include two that test orientation to time and a three word delayed recall test, very similar to the MMSE items in the ‘six item screener’ selected on theoretical grounds (Callahan *et al.*, 2002). The item difficulties suggested that the resulting scales were discriminating largely among the most impaired 10% of the population, a satisfactory distribution for a dementia screening assessment. In one of the two test data sets (the 10/66 DRG pilot study data set) the gold standard dementia diagnosis was entirely independent of the CSI-D assessment, while in the other (the Cuba population-based study data set) clinicians made their gold standard diagnosis after CSI-D administration it had not, at that stage, been scored or formally incorporated into the computerised survey dementia diagnoses. Thus verification bias was largely avoided.

In this context, the performance of the brief CSI-D is highly encouraging, indeed remarkably similar to that of the full CSI-D in the 10/66 pilot study (Prince *et al.*, 2003). The brief CSI-D would comfortably match the criteria previously laid down for a measure that could be completed within 5 min (Brodaty *et al.*, 2006), yet would at least match the performance of the full MMSE in the primary care setting (Wind *et al.*, 1997). It is clear that the inclusion of an informant interview adds to the validity of the brief CSI-D, as was the case with the full CSI-D (Prince *et al.*, 2003). Of course, the informant scale may be inconvenient to complete if a suitable informant does not attend with the person to be tested. Under such circumstances, a conservative strategy supported by our data is that a score of four or less on the cognitive scale should be regarded as highly suggestive of dementia, whereas a score of seven or more renders the diagnosis highly improbable. For those scoring five or six, an additional informant interview could be particularly helpful, and a reassessment after several months might also be indicated. A combined score of four or less, after subtraction of the informant score from the cognitive score would then be highly suggestive of dementia.

The main limitations of this exercise are first that the brief CSI-D was not administered in its brief form, but, rather, interspersed with the remaining items from the full scale, and, second, that the administration was carried out at home, by research workers, rather than in primary care by non-specialist healthcare professionals. Either or both of these factors may limit the generalisability of our findings. Therefore, a further formal validation needs to be carried out in primary care using the brief form of the cognitive and informant assessments. Primary care professionals may need brief training in its administration and scoring. A suitable base population for the formal validation, to reflect likely patterns of use would be any older users of primary care services that the health professionals might feel to be possibly suffering from dementia, as used successfully in some previous studies (Cooper *et al.*, 1992).

## Conclusions

A brief version of the full Community Screening Instrument for Dementia (CSI-D) appears to share the very favourable screening properties of the full assessment, despite considerable abbreviation (7 versus 32 cognitive test items, and 6 versus 26 informant items). The robust cross-cultural measurement properties of the parent instrument also seem to be preserved. The feasibility and validity of the brief version of the CSI-D still need to be established in the context of routine primary care practice.

## Declarations

### Funding

The 10/66 Dementia Research Group's research has been funded by the Wellcome Trust Health Consequences of Population Change Programme (GR066133 – Prevalence phase in Cuba and Brazil; GR08002 – Incidence phase in Peru, Mexico, Argentina, Cuba, Dominican Republic, Venezuela and China), the World Health Organisation (India, Dominican Republic and China), the US Alzheimer's Association (IIRG – 04 – 1286 – Peru, Mexico and Argentina) and FONDACIT (Venezuela). The Rockefeller Foundation supported our recent dissemination meeting at their Bellagio Centre. Alzheimer's Disease International has provided support for networking and infrastructure.

### Authors' contributions

All of the authors worked collectively to develop the protocols and methods described in this paper. Martin Prince led the research group and Cleusa Ferri acted as research coordinator. Juan Llibre Rodriguez (Cuba), Daisy Acosta (Dominican Republic), Mariella Guerra (Peru), Aquiles Salas (Venezuela), Ana Luisa Sosa (Mexico), KS Jacob (Vellore, India), Joseph D Williams (Chennai, India) and Yueqin Huang (China) were principal investigators responsible for the field work in their respective countries. Martin Prince wrote the first draft and conducted the analyses. Other authors reviewed the manuscript, provided further contributions and suggestions. All authors read and approved the final manuscript.

**Key Points**

- The cognitive and informant scales of the Community Screening Instrument for Dementia can be considerably abbreviated (to seven and six items respectively), while seemingly retaining the excellent culture-fair screening properties of the parent instrument
- The two brief scales have very strong hierarchical measurement properties
- The brief version should, in principle, be suitable for use by non-specialist health workers in primary care settings, but its feasibility and validity needs to be formally established in that context.

### Conflict of interest

The 10/66 Dementia Research Group works closely with Alzheimer's Disease International, which is a non-profit federation of 77 Alzheimer associations around the world. Daisy Acosta is currently Chair of Alzheimer's Disease International. ADI is committed to strengthening Alzheimer associations worldwide, raising awareness regarding dementia and Alzheimer's disease, and advocating for more and better services for people with dementia and their caregivers. ADI is supported in part by grants from GlaxoSmithKline, Novartis, Lundbeck, Pfizer and Eisai.

Kathleen Hall led the development of the Community Screening Instrument for Dementia.

## Appendix

# The 10/66 Dementia Research Group, part of Alzheimer's Disease International, is a collective of researchers from the developing and developed regions of the world. A full list of members with contact details can be found at http://www.alz.co.uk/1066. The following members of the 10/66 Group participated as investigators in the pilot phase of this project.

**Co-ordinating Centre**: Prof. Martin Prince, Institute of Psychiatry, London; Prof. John Copeland, University of Liverpool, Dr Michael Dewey, Nottingham

**10/66 India Bangalore**: Dr. Mathew Varghese and Dr. Srikala Bharath, NIMHANS, Bangalore; Chennai (SCARF) - Ms. Latha Srinivasan, Dr. R. Thara, Schizophrenia Research Foundation; Chennai (VHS) - Mr Ravi Samuel, Dr. E.S. Krishnamoorthy, Voluntary Health Services; Goa – Dr. Vikram Patel, Dr. Amit Dias, Sangath, Goa; Hyderabad – Dr. K. Chandrasekhar, Dr. M. Ajay Verma, Heritage Hospitals; Thrissur – Asst. Prof KS Shaji, Prof. K Praveen Lal, Medical College, Thrissur; Vellore – Prof KS Jacob, Dr. Arockia Philip Raj, Christian Medical College.

**10/66 China and SE Asia China (Beijing)**: Prof. Li Shuran, Dr. Jin Liu, Beijing University; China (Hong Kong SAR) – Prof. Linda Lam, Dr. Teresa Chan, Chinese University of Hong Kong; Taiwan (Taipei) – Dr. Shen-Ing Liu, Mackay Memorial Hospital, Prof. P. K. Yip, National Taiwan University Hospital.

**10/66 Latin America and Caribbean Argentina (Buenos Aires)**: Dr. Raúl Luciano Arizaga, Hospital Santojanni (GCBA). Dr. Ricardo F. Allegri, Hospital Zubizarreta (GBCA Y CONICET); Brazil (Sao Paulo) – Dr. Marcia Scazufca, Dr. Paulo Rossi Menezes, Universidade de São Paulo; Brazil (Botucatu) – Dr. Ana Teresa de A. R. Cerqueira, Botucatu Medical School – UNESP; Brazil (São Jose do Rio Preto) – M. Cristina O. S. Miyazaki,Neide A. Micelli Domingos, FAMERP Medical School; Chile (Santiago/ Concepción,/Valparaíso) – Dr. Patricio Fuentes G. Hospital Del Salvador, Santiago, Dra. Pilar Quiroga L. Universidad de Concepción, Concepción; Cuba (Havana) - Dr. Juan de J Llibre Rodríguez, Dr. Hector Bayarre Vea, Facultad de Medicina ‘Finlay-Albarran’, Universidad Medica de la Habana; Dominican Republic (Santo Domingo) – Dr Daisy Acosta, Universidad Nacional Pedro Henriquez Ureña (UNPHU), Lic. Guillermina Rodriguez, Asociación Dominicana de Alzheimer (ADA); Guatemala (Guatemala City) – Dr. Carlos A. Mayorga Ruiz, Dr. Mario Luna de Floran; Mexico (Mexico City) – Dra. Ana Luisa Sosa, Dra. Yaneth Rodriguez Agudelo, National Institute of Neurology and Neurosurgery; Mexico (Guadalajara) – Dr. Genaro G. Ortiz Lab Desarrollo/Envejecimiento. CIBO/IMSS. Dra. Elva D. Arias-Merino, Gerontología, Universidad de Guadalajara; Panama (Panama City) - Dr. Gloriela R. de Alba, Paitilla Medical Center Hospital, Dr. Gloria Grimaldo, Santa Fe Hospital; Peru (Lima) – Dr. Mariella Guerra. Instituto Nacional de Salud Mental ‘ Honorio Delgado-Hideyo Noguchi’, Universidad Peruana Cauetano Heredia, M. Víctor González, Instituto Peruano de Seguridad Social – ESSALUD; Uruguay (Montevideo) – Dr. Roberto Ventura, Dr. Nair Raciope, University of Uruguay; Venezuela (Caracas) – Dr. Aquiles Salas, Universidad Central de Venezuela, Faculty of Medicine, Dr. Ciro Gaona, Fundación Alzheimer's Venezuela.

**10/66 Africa**: Nigeria (Anambra) – Dr. Richard Uwakwe, Nnamdi Azikiwe University Teaching Hospital.

**10/66 Russia**: Moscow (Russia) – Prof Svetlana Gavrilova, Dr Grigory Jarikov. Alzheimer's Disease Research Center, Mental Health Research Center of Russian Academy of Medical Sciences.

The brief community screening instrument for dementia (CSI-D)

**Table:**

Name of person being assessed	
Age in years	
Sex	Male
	Female
Highest completed level of education	None
	Minimal
	Completed primary
	Completed secondary
	Completed tertiary

Now I am going to tell you three words and I would like you to repeat them after me

Boat

House

Fish

Repeat the three words, up to a maximum of six times. or until the person has remembered them all correctly. Then say:

Very good, now try to remember these words because I will be asking you later

**Table:**

*Question*	Incorrect (score 0)	Correct (score 1)
*(Interviewer points to their elbow)* What do we call this?		
What do you do with a hammer? *Acceptable answer ‘To drive a nail into something’*		
Where is the local market/local store?		
What day of the week is it?		
What is the season?		
Please point first to the window and then to the door		
Do you remember the three words I told you a few minutes ago?		
Boat		
House		
Fish		
TOTAL SCORE (MAXIMUM = 9)		
Probable dementia	0–4	
Possible dementia (check informant score)	5–6	
Normal	7–9	

I would like to ask a few brief questions about your xxxxx's activities these days.

**Table:**

Questions	No = 0	Yes = 1
Has there been a general decline in her mental functioning?		
Have you noticed a change in her ability to think and reason?		
Does she often forget where she has put things?		
Does she sometimes forget what happened the day before?		
Does she sometimes forget where she is?		
Does she have difficulty dressing (misplacing buttons, putting clothes on in the wrong order or in the wrong way)?		
- if because of physical disability then code 0		
		
TOTAL SCORE (MAXIMUM = 6)		
